# Pathogenic variants of Valosin-containing protein induce lysosomal damage and transcriptional activation of autophagy regulators in neuronal cells

**DOI:** 10.1111/nan.12818

**Published:** 2022-05-15

**Authors:** Veronica Ferrari, Riccardo Cristofani, Maria E. Cicardi, Barbara Tedesco, Valeria Crippa, Marta Chierichetti, Elena Casarotto, Marta Cozzi, Francesco Mina, Mariarita Galbiati, Margherita Piccolella, Serena Carra, Thomas Vaccari, Angele Nalbandian, Virginia Kimonis, Tyler R. Fortuna, Udai B. Pandey, Maria C. Gagliani, Katia Cortese, Paola Rusmini, Angelo Poletti

**Affiliations:** 1 Dipartimento di Scienze Farmacologiche e Biomolecolari, Centre of Excellence on Neurodegenerative Diseases, Università degli Studi di Milano, Milan; 2 Department of Neuroscience, Weinberg ALS Center, Vickie and Jack Farber Institute for Neuroscience, Thomas Jefferson University, Philadelphia, PA, USA; 3 Unit of Medical Genetics and Neurogenetics, Fondazione IRCCS – Istituto Neurologico Carlo Besta, Milan, Italy; 4 Department of Biomedical, Metabolic and Neural Sciences, University of Modena and Reggio Emilia, Modena, Italy; 5 Dipartimento di Bioscienze, Università degli Studi di Milano, Milan, Italy; 6 Department of Pediatrics, University of California, Irvine, CA, USA; 7 Department of Pediatrics, Children’s Hospital of Pittsburgh, University of Pittsburgh Medical Center, Pittsburgh, PA, USA; 8 Department of Human Genetics, Graduate School of Public Health, University of Pittsburgh, Pittsburgh, PA, USA; 9 Department of Experimental Medicine (DIMES), Cellular Electron Microscopy Lab, University of Genoa, Genova

**Keywords:** ALS, lysosome, neurodegeneration, p97, PQC, TFE3

## Abstract

**Aim:**

Mutations in the valosin-containing protein (*VCP*) gene cause various lethal proteinopathies that mainly include inclusion body myopathy with Paget’s disease of bone and frontotemporal dementia (IBMPFD) and amyotrophic lateral sclerosis (ALS). Different pathological mechanisms have been proposed. Here, we define the impact of VCP mutants on lysosomes and how cellular homeostasis is restored by inducing autophagy in the presence of lysosomal damage.

**Methods:**

By electron microscopy, we studied lysosomal morphology in VCP animal and motoneuronal models. With the use of western blotting, real-time quantitative polymerase chain reaction (RT-qPCR), immunofluorescence and filter trap assay, we evaluated the effect of selected VCP mutants in neuronal cells on lysosome size and activity, lysosomal membrane permeabilization and their impact on autophagy.

**Results:**

We found that VCP mutants induce the formation of aberrant multilamellar organelles in VCP animal and cell models similar to those found in patients with *VCP* mutations or with lysosomal storage disorders. In neuronal cells, we found altered lysosomal activity characterised by membrane permeabilization with galectin-3 redistribution and activation of PPP3CB. This selectively activated the autophagy/lysosomal transcriptional regulator TFE3, but not TFEB, and enhanced both SQSTM1/p62 and lipidated MAP1LC3B levels inducing autophagy. Moreover, we found that wild type VCP, but not the mutants, counteracted lysosomal damage induced either by trehalose or by a mutant form of SOD1 (G93A), also blocking the formation of its insoluble intracellular aggregates. Thus, chronic activation of autophagy might fuel the formation of multilamellar bodies.

**Conclusion:**

Together, our findings provide insights into the pathogenesis of VCP-related diseases, by proposing a novel mechanism of multilamellar body formation induced by VCP mutants that involves lysosomal damage and induction of lysophagy.

## INTRODUCTION

Valosin-containing protein (VCP) is a ubiquitously expressed ATPase associated with diverse cellular activities (AAA^+^) [1,2], which is critical in cellular homeostasis. VCP contains two ATPase domains: the D1 site energises VCP homohexamer formation, whereas the D2 site supports VCP biological functions [3]. VCP N- and C-terminals interact with adaptors and cofactors [4]. VCP is mainly a soluble cytoplasmic protein but partly associates with the membranes of several organelles (e.g., the endoplasmic reticulum [ER], Golgi apparatus, mitochondria and some endosomes and lysosomes) or localises into the nucleus [5–9]. Nuclear VCP is involved in protein quality control (PQC) and chromatin remodelling processes [10–12], whereas cytoplasmic VCP participates in unfolded protein degradation and in organelle formation and degradation. VCP substrate recognition is mediated by cofactors (e.g., UBX domain-containing 1 [UBXD1], phospholipase A2 activating protein [PLAA] and UFD1-NPL4 complex) that mark substrates (i.e., misfolded proteins), leading to their ubiquitination or deubiquitination and subsequent VCP-mediated segregation from membranes, macromolecular complexes or protein aggregates for ubiquitin proteasome system (UPS)-mediated degradation [13–15]. VCP also controls damaged DNA repair [16], regulates damaged lysosomes and mitochondria degradation [9,17] and cooperates in autophagy [18,19].

*VCP* mutations cause inclusion body myopathy with early-onset Paget disease and frontotemporal dementia (IBMPFD), also known as multisystem proteinopathies (MSPs), familial forms of amyotrophic lateral sclerosis (fALS) and/or frontotemporal dementia (FTD) and Parkinson’s disease (PD) [20–23]. MSPs are age-related degenerative diseases involving muscle, bone and brain that are associated with mutations of *VCP* and other less common genes [23–26]. The *VCP* variants alter ATP binding, thus modifying VCP conformation and binding with some cofactors [27–29]. This perturbs the PQC system, altering the handling and clearance of misfolded proteins, and the clearance of damaged organelles [16,30–33]. Indeed, VCP mutants cause TAR-DNA binding protein-43 (TARDBP/TDP-43) mislocalisation and aggregation, typical findings in sporadic ALS (sALS) and FTD [21].

Wild type (WT) and mutant VCP affect autophagy, but their role in this process is still debated. VCP regulates the lipid phosphatidylinositol-3-phosphate (PtdIns3P) via beclin1 (BECN1) containing phosphatidylinositol 3-kinase (PtdIns3K) complex and ataxin 3 (ATXN3) deubiquitinase activity, which stabilises BECN1 promoting PtdIns3K complex formation and activity. VCP also promotes biogenesis of autophagosomes, and its inhibition alters initiation of autophagy [34]. Both *VCP* silencing and pathogenic *VCP* variants lead to impairment of autophagy with an accumulation of sequestosome-1 (SQSTM1/p62) and the activated form of microtubule-associated proteins 1A/1B light chain 3B (MAP1LC3B) [35,36]. However, some reports have pointed to the opposite effect of VCP in autophagy: the inhibition of VCP is associated with SQSTM1/p62 degradation that is reversed by blocking autophagy [19]. Despite this, it is well established that VCP-regulated autophagy is essential for lysosomal dynamics and damaged lysosomal clearance (lysophagy). In cooperation with specific cofactors (PLAA, UBXD1, YOD1 deubiquitinase), VCP assists galectin-3 (LGALS3) activity in the removal of damaged lysosomes. VCP selectively removes K48-ubiquitinated substrates from a subset of damaged lysosomes promoting their clearance. This activity is lost by VCP mutants [9]. Moreover, *Vcp* knockout in mouse muscles induces a necrotic myopathy with damaged lysosomes and LGALS3 upregulation, inducing autophagy via transcription factor EB (TFEB) activation [37]. TFEB is activated by treatment with L-leucyl-L-leucine methyl ester (LLOMe), which induces lysosomal membrane permeabilization (LMP). After a recovery phase, LLOMe-activated TFEB remains localised in the nucleus when *VCP* is inactivated or VCP mutants are expressed. This suggests that WT and mutant VCP modulate TFEB activity. Notably, lysosomal damage with LGALS3 and LAMP1-positive puncta and TFEB activation is detectable in muscles of the VCP-disease mouse model. However, in this model, TFEB redistribution occurs only at very late stages of disease (1-year-old mice), suggesting that TFEB does not play a direct role in the early stages of VCP-related diseases.

Therefore, it is established that WT VCP and its mutants have different effects on the recovery from induced lysosomal damage. Nevertheless, it is still unclear whether VCP mutants directly induce lysosomal damage in basal conditions or what mechanisms activate lysophagy in the early stages of the disease.

Here, we studied whether VCP mutants linked to fALS/FTD impact lysosomal functions and whether this results in the modulation of basal autophagy in affected motoneurons. We found that VCP mutants directly affect lysosomal dynamics and specifically impact autophagic activity via the activation of transcription factor binding to IGHM enhancer 3 (TFE3) but not activation of TFEB. TFE3 activation is induced by dephosphorylation via protein phosphatase 3 catalytic subunit beta (PPP3CB). Its activation enhances SQSTM1/p62 levels and MAP1LC3B lipidation, with potentiation of autophagic flux. This enhances lysophagy, clearing damaged lysosomes in response to mutant VCP expression. We also found that WT VCP, but not mutant VCPs, counteracts lysosomal damage induced either by trehalose or by mutant G93A SOD1, preventing intracellular aggregate formation.

Protective and detrimental activities were linked to the intracellular response to mutant VCP expression, eventually dysregulating the response of the PQC system.

## MATERIALS AND METHODS

### Chemicals

The following chemicals were used: D-(+)-trehalose dihydrate (trehalose) (100mM, Sigma-Aldrich, T9531); NH_4_Cl (4mM, Euroclone, EMRO895009); Chloroquine/CQ (25 μM, Sigma-Aldrich, C6628).

### Plasmids and siRNAs

pFLAG-VCP WT and pFLAG-VCP R155H encoding for human WT VCP and the R155H mutant were obtained from Prof. J.P. Taylor (Cell & Molecular Biology Department, St. Jude Children’s Research Hospital, Memphis, TN, USA). pFLAG-VCP R191Q was obtained by single-nucleotide mutagenesis from pFLAG-VCP WT (Eurofins Genomics), replacing guanine with adenosine to convert the Arginine 191 into Glutamine.

p6xHIS-VCP WT, p6xHIS-VCP R155H and p6xHIS-VCP R191Q were obtained by excising the FLAG tag from pFLAG-VCPs with HindIII/EsprI and inserting an in-frame 6xHIS sequence.

pEGFP-LGALS3 is from Prof. M. A. Jäättelä (Danish Cancer Society Research Center, Copenhagen, Denmark).

pEGFP-N1-TFEB and pEGFP-N1-TFE3 were obtained from Prof. Shawn Ferguson (Addgene plasmids #38119 and #38120, respectively; http://n2t.net/addgene:38120; RRID:Addgene_38120; http://n2t.net/addgene:38119; RRID:Addgene_38119 [38]).

pLAMP1-GFP was obtained from Prof. Ron Vale (Addgene plasmid #16290; http://n2t.net/addgene:16290; RRID:Addgene_16290 [2]).

pSOD1 WT and pSOD1 G93A were obtained from Dr Caterina Bendotti (Mario Negri Institute, Milano, Italy).

pCDNA3 is from Life Technologies (V790–20), and pEGFPN1 from Clontech (U55762).

The following siRNA duplex was used for silencing mPPP3CB expression: siRNA sense: 5′UGACAGAAAUGUUGGUAAAUU3′ and antisense: 5′UUUACCAA CAUUUCUGUCAUU3′ (Dharmacon); the non-targeting siRNA sense: 5′UAGCGACUAAACACAUCAAUU3′ and antisense: 5′UUGA UGUGUUUAGUCGCUAUU3′ (Dharmacon).

### Cell cultures and transfections

Neuroblastoma spinal cord (NSC-34) cells are mouse motoneuron immortalised cells routinely used in our laboratory. NSC-34 cells were maintained as described in [39] and plated as follows: for real-time quantitative polymerase chain reaction (RT-qPCR), western blotting (WB), filter trap assay (FTA), lysosomal activity test and lysosomal acidification status in 12-well plates at 90,000cells/ml; for immunofluorescence (IF), stimulated emission depletion (STED), Galectin puncta assay and lysosomal size and number analysis on 13-mm coverslips in 24-well plates at 70,000cells/ml. Plasmids were transfected for 48 h using Lipofectamine3000^®^ Transfection Reagent (Invitrogen, ThermoFisher-Scientific, L3000–015) following the manufacturer’s protocol.

siRNAs were transfected for 72 h using Lipofectamine2000 (Life-Technologies, 11668019) with 40 pmol in 12-well plates and 20 pmol in 24-well plates, following the manufacturer’s protocol.

### Animals

Transgenic R155H VCP mice were generated on 129/SvEv [40] and backcrossed more than 6 times with the C57BL/6 strain so that the majority (>98%) of the genetic background of the generated mice was of C57BL/6 origin. Littermates were used for experiments and maintained as previously described [40].

For conditional ubiquitous expression in *Drosophila*, UAS-VCP (WT and mutant R152H) lines [41] were crossed with the inducible driver, TubGS-Gal4. Day 1 adults from the F1 progeny were collected every 24 h and moved to standard media mixed with 20mM RU486 as described in [42,43]. The F1 progeny adults were aged for 10 days at 25°C when the thorax was dissected out and fixed in Davidson’s fixative (Electron Microscopy Sciences; #64133–10).

### RT-qPCR

NSC-34 cells were transfected with target siRNA or with plasmids listed in Table S2. Total RNA was extracted using Tri-Reagent (Sigma-Aldrich, T9424) following the manufacturer’s protocol. Before reverse transcription (RT) of RNA samples, the RNA concentration and quality were tested measuring the ratio of the absorbance at 260/280 nm and 260/230 nm for each sample (data are included in Tables S3 and S4); 1 μg per sample was treated with DNase and reverse transcribed using the High-Capacity cDNA Reverse Transcription Kit (ThermoFisher-Scientific, 4368814).

RT-qPCR was performed using the CFX96 Real-Time System (Bio-Rad Laboratories), the iTaq SYBR Green Supermix (Bio-Rad Laboratories, 1725124), and with a final concentration of 500 nM of primers. Data were normalised using Rplp0. The experiments were performed with 4 independent samples (*n* = 4).

### WB and FTA

NSC-34 were transfected with plasmids listed in Table S2.

Cells were harvested, centrifuged, resuspended, and lysed as described in Rusmini et al. [44]. Total protein content was quantified with bicinchoninic acid (BCA) assay (Cyanagen, PRTD1).

For WB analysis, 15-μg proteins/samples added with β-mercaptoethanol and SDS to denature and linearize proteins were loaded on a polyacrylamide gel. After electrophoresis, proteins were transferred with Trans-Blot Turbo (Bio-Rad Laboratories, 1704150) for 40 min at 25 V at RT on a 0.45-μm nitrocellulose membrane (Bio-Rad Laboratories, 1620115).

For FTA, 6-μg proteins/samples were filtered on a 0.22-μm cellulose-acetate membrane (Whatman, 100404180) using a Bio-Dot SF Microfiltration Apparatus (Bio-Rad Laboratories, 1703938). Then proteins were fixed with 20% methanol solution.

To quantify cytoplasmic and nuclear levels of TFEB and TFE3, NSC-34 cells were plated in 6-well plates at 90,000cells/ml and transfected with plasmids listed in Table S2 or treated for 24 h with trehalose. Cells were harvested, centrifuged and lysed, and nuclear-cytoplasmic fractions were isolated as described [44,45]. Antibodies used for WB assay and FTA are listed in Table S1. Immunoreactivity was detected with enhanced chemiluminescent (ECL) detection reagent (Westar Antares, Cyanagen XLS0142) acquiring images with Chemidoc XRS System (Bio-Rad Laboratories, 1708265). Densitometric quantification was performed using Image Lab Software, version5.2.1 (Bio-Rad Laboratories).

### IF analysis

NSC-34 were transfected with plasmids listed in Table S2. To induce chemical lysosome damage, cells were treated with trehalose for 2, 6 or 18 h. Cells were fixed with 4% paraformaldehyde solution, permeabilized for 15 min using 10% Triton X-100 in phosphate-buffered saline (PBS) solution, incubated for 1 h at RT in blocking solution and then with primary antibody overnight at 4°C followed by 1 h at RT with secondary antibody. Nuclei were stained with 4′,6-diamidino-2-phenylindole (DAPI) (1:10,000 in PBS). Coverslips were mounted using Mowiol^®^ 4–88 (Merck-Millipore, 475904). Images were acquired with Axiovert 200 microscope (Zeiss, Oberkochen, Germany). TFEB and TFE3 nuclear intensities were quantified with ImageJ software as described in Rusmini et al. [44]. The size of SQSTM1/p62 and MAP1LC3B puncta of aggregate-like structures and their cellular distribution at single-cell resolution were quantified using an ImageJ macro (AggreCount [46]) for unbiased analyses of cellular aggregate-like structures. Antibodies used for IF assay are listed in Table S1. To quantify lysosomal number and size, images were acquired with LSM510 Meta system confocal microscope (Zeiss) and were processed with the Aim 4.2 software (Zeiss). Z-stacks of 11 randomly selected fields per condition (see figure legends for details) were acquired, and lysosomal number and volume were quantified with Arivis Vision4D software.

### STED microscopy

NSC-34 were transfected with plasmids listed in Table S2. Cells were fixed, permeabilized and processed as described for IF assay. Images were taken with a Leica TCS SP8 STED 3× microscope with 3 depletion lines (592, 660 and 775 nm), with HC PL APO 100×/1.40 oil objective, acquired through the Software Leica LAS X and processed using ImageJ (version 1.51).

### Galectin puncta assay

NSC-34 were transfected with plasmids listed in Table S2. To induce chemical lysosome damage, cells were treated with trehalose for 2, 6 or 18 h; 48 h from transfection, cells were processed as described for IF assay. Cells with >3 EGFP-LGALS3 puncta were manually quantified in 3 randomly selected fields per sample (see figure legends for details) condition using a PL 20× eyepiece with graticules (100×10 mm in 100-grid divisions) as described in Rusmini et al. [44]. 3 samples per condition were analysed. Cells expressing green fluorescent protein (GFP) were counted in the same field. The ratio between cells with >3 EGFP-LGALS3 puncta and transfected cells was calculated on each field of view and statistical analysis was performed.

### Lysosomal activity

Lysosomal enzyme activity was measured by cytometer analysis in NSC-34 transfected with plasmids listed in Table S2. Cells were incubated in high glucose medium with 0.5% of fetal bovine serum (FBS) and self-quenched substrate (PromoCell, PK-CA577-K448) for 1 h as described by the manufacturer and then collected, washed twice in 1-ml assay buffer, resuspended in PBS and analysed on a NovoCyte flow cytometer (Acea Biosciences, Inc). Mean fluorescence intensity (MFI) of the self-quenched substrate was recorded from 10,000 cells for each sample (*n* = 6).

### Lysosomal acidification status

Relative lysosomal acidification status was measured in NSC-34 transfected with plasmids listed in Table S2 and treated with trehalose for different time periods (from 2 to 18 h). Next, the cells were incubated with 100nM of lysosomotropic probe LysoTracker Green DND-26 (ThermoFisher Scientific, L7526) for 30 min. The cells were collected, resuspended in 4% FBS in PBS and analysed with NovoCyte flow cytometer (Acea Biosciences, Inc.). The mean LysoTracker fluorescence intensity was recorded from 50,000 cells for each sample (*n* = 4).

### EM analysis

NSC-34 cells were plated at 90,000 cells/ml in a 2-well Nunc^®^Lab-Tek^®^Chamber Slide™ system (Nunc, C6682) and transfected with plasmids listed in Table S2. Cells were then washed in 0.1M cacodylate buffer and fixed in 0.1M cacodylate buffer containing 2.5% glutaraldehyde (Electron Microscopy Science, Hatfield, PA, USA) for 1 h at RT. Cells were post-fixed in 1% osmium tetroxide for 2 h and 1% aqueous uranyl acetate for 1 h, dehydrated through a graded ethanol series and flat embedded in resin (Poly-Bed; Polysciences, Inc., Warrington, PA) for 24 h at 60°C. Ultrathin sections (50 nm) were cut parallel to the substrate and counterstained with 5% uranyl acetate in 50% ethanol; 30 images from each sample were collected at 25,000× magnification for measurements of lysosomal diameters.

For ultrastructural analysis of animal tissues, W1118, WT VCP and mutant R152H VCP adult *Drosophila* (Day 10) muscle thoraces were placed in cacodylate buffer containing 2.5% glutaraldehyde for 24 h, post-fixed in 1% osmium tetroxide and 1% uranyl acetate for 1 h each, dehydrated through a graded ethanol series and with propylene oxide for 1 h. Next, tissues were embedded in epoxy resin (PolyBed; Polysciences, Inc.) overnight at 42°C and 2 days at 60°C. Ultrathin sections (50 nm) were observed with a HITACHI 7800 120Kv electron microscope (Hitachi, Tokyo, Japan), and digital images acquired with Megaview3 CCD camera and RADIUS software (EMSIS, Germany). Quantification of lysosome size was performed as described for NSC-34 cells. Post-processing on digital images was performed with Adobe Photoshop2021 and Adobe Illustrator2021.

For analysis of quadriceps muscle from WT and knock-in mutant VCP R155H 10-month-old mice, 3 WT control mice and 8 VCP R155H mutant littermates were fixed for 24 h at 4°C in 0.1M PBS solution added with 4% paraformaldehyde and 0.1% glutaraldehyde. Tissue samples were fixed for 1 h at 4°C in 1% osmium solution and subsequently dehydrated in ethanol. Ultrathin (60, 80 nm) sections were cut, stained and incubated as described in Nalbandian et al. [47]. Electron micrographs were taken with a Gatan UltraScan US1000 digital camera and analysed for architectural differences and lipid accumulation.

### Statistical analysis

**FIGURE 1:**
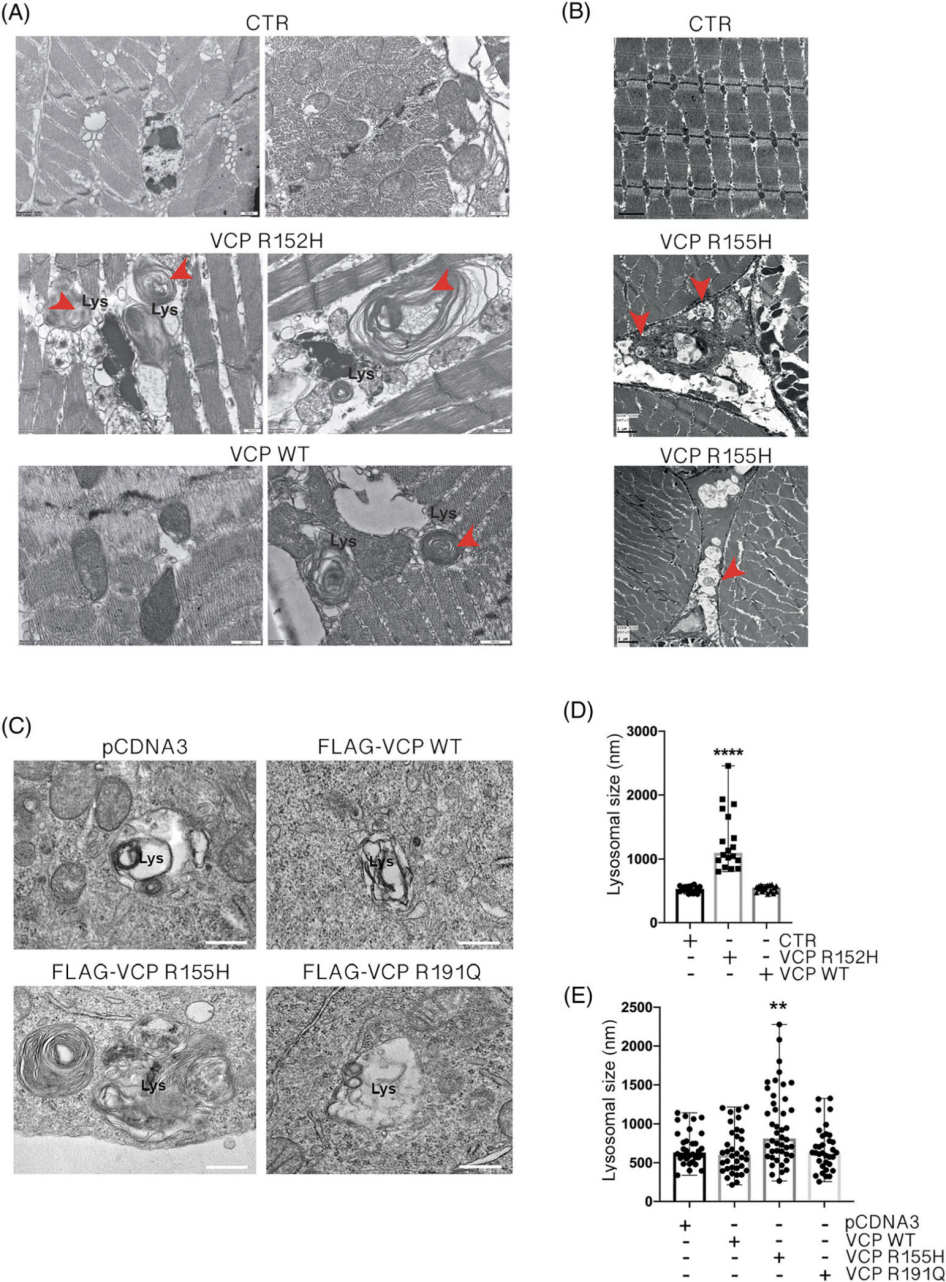
Abnormal lysosomal structures in tissues and cells expressing mutant valosin-containing proteins (VCPs). (A) Electron microscopy (EM) analysis of lysosomes in thoracic muscle of *Drosophila melanogaster* expressing either wild type (WT) (CTR) or R152H mutation. Scale bar, 600 nm. Red arrows evidence multilamellar bodies (MLBs). (B) EM analysis of lysosomes in quadriceps muscles of control (CTR) and transgenic R155H VCP mice. Scale bar, 1 or 2 μm. Red arrows evidence MLBs. (C) EM analysis of lysosomes in NSC-34 expressing WT or mutants FLAG-VCPs. Scale bar, 200 nm. (D) The bar graph represents lysosomal diameters measured on EM analysis of *D. melanogaster* expressing either WT (CTR) or R152H mutation; 32–42 lysosomes were analysed for each condition (Kruskal–Wallis test followed by Dunns multiple comparison test; ****p < 0.0001). (E) The bar graph represents lysosomal diameters measured on EM analysis of NSC-34 expressing WT or mutants FLAG-VCPs; 33–42 lysosomes were analysed for each condition (Kruskal–Wallis test followed by Dunns multiple comparison test; **p < 0.01)

The data in Figure 1 are presented as median–range as they do not follow a Gaussian distribution; all the other data are presented as mean SD. For a variable in two groups, an unpaired *t* test was used. In the presence of 3 or more groups, one-way analysis of variance (ANOVA) was used followed by a post hoc test as described in the legends. An unpaired *t* test with Welch’s correction was used when the SDs were significantly different as determined by Bartlett’s test (see figure legends for details). Statistical analysis was performed using two-way ANOVA to compare the effect of 2 independent variables when 3 or more groups were present. When two-way ANOVA was significant, a post hoc test was performed (see figure legends for details). *P* values < 0.05 were considered statistically significant. All the analyses were undertaken with the PRISM (version 8.2.1.) software.

## RESULTS

### VCP mutants aggregate and induce lysosomal damage

VCP-patient muscles are characterised by degeneration and the presence of mutant VCP inclusions [48]. We noted some published electron microscopy (EM) images of these skeletal muscles showing abnormal multilamellar structures, not originally described by Watts et al. [48], that morphologically resembled the membranous concentric bodies typically found in lysosomal storage disorders (LSDs) [49,50]. In LSDs, lysosomes mostly containing undegraded phospholipids and cholesterol accumulate forming multilamellar bodies (MLBs). The formation of MLBs might be a consequence of an impairment in autophagy. Indeed, initially single or multiple foci of lamellae appear within an autophagic vacuole and then transform into multilamellar structures [51]. To evaluate whether these multilamellar structures are a specific feature of VCP disease in muscle, we analysed EM tissue samples derived from flies (*Drosophila melanogaster*) expressing WT or R152H Vcp under the control of a conditional ubiquitous driver (Tub-GS) and from mutant mice expressing the R155H Vcp. The thoracic muscles from VCP flies (Figure 1A) and quadriceps muscles from mice (Figure 1B) showed MLBs (evidenced by arrows) in large areas of the tissues. We thus investigated whether MLBs are formed only as a chronic response to the stressor activities of mutant VCP, as they are present in the late stages of the disease, or whether they could be acutely induced in overexpressing cells. Therefore, with the use of transient transfection, we established VCP motoneuronal cell models to analyse whether MLBs appear in EM under acute conditions. Two ALS-associated VCP mutants, R155H VCP and R191Q VCP, were expressed in the immortalised murine motoneuronal cell line NSC-34, widely used as a bona fide neuronal model in ALS [52–54]. Figure 1C shows that cells expressing VCP mutants, particularly R155H VCP, have abnormal lysosome morphology, very similar to those seen in the muscles of the animal models (Figure 1A,B) and in skeletal muscle of VCP patients [48]. MLBs in R191Q VCP/NSC-34 cells were less pronounced compared to those found in R155H VCP/NSC-34 cells. Several abnormal membranes were also retained in the lumen of these lysosomes that were not present in NSC-34 cells overexpressing WT VCP. Unfortunately, their large variability in size and form prevented us from performing quantification of these structures. Thus, we determined whether the size of lysosomes from which the MLBs are derived is increased in the presence of the mutated forms of VCP. Very interestingly, we found a robust increase of lysosomal size both in the muscle of R152H VCP flies (Figure 1D) and in R155H VCP/NSC-34 cells (Figure 1E), compared with their respective controls. A trend towards increased lysosome size was noted also in R191Q VCP/NSC-34 cells, but this did not reach statistical significance, suggesting that this VCP mutant is less potent at producing an acute alteration of lysosomes.

**FIGURE 2:**
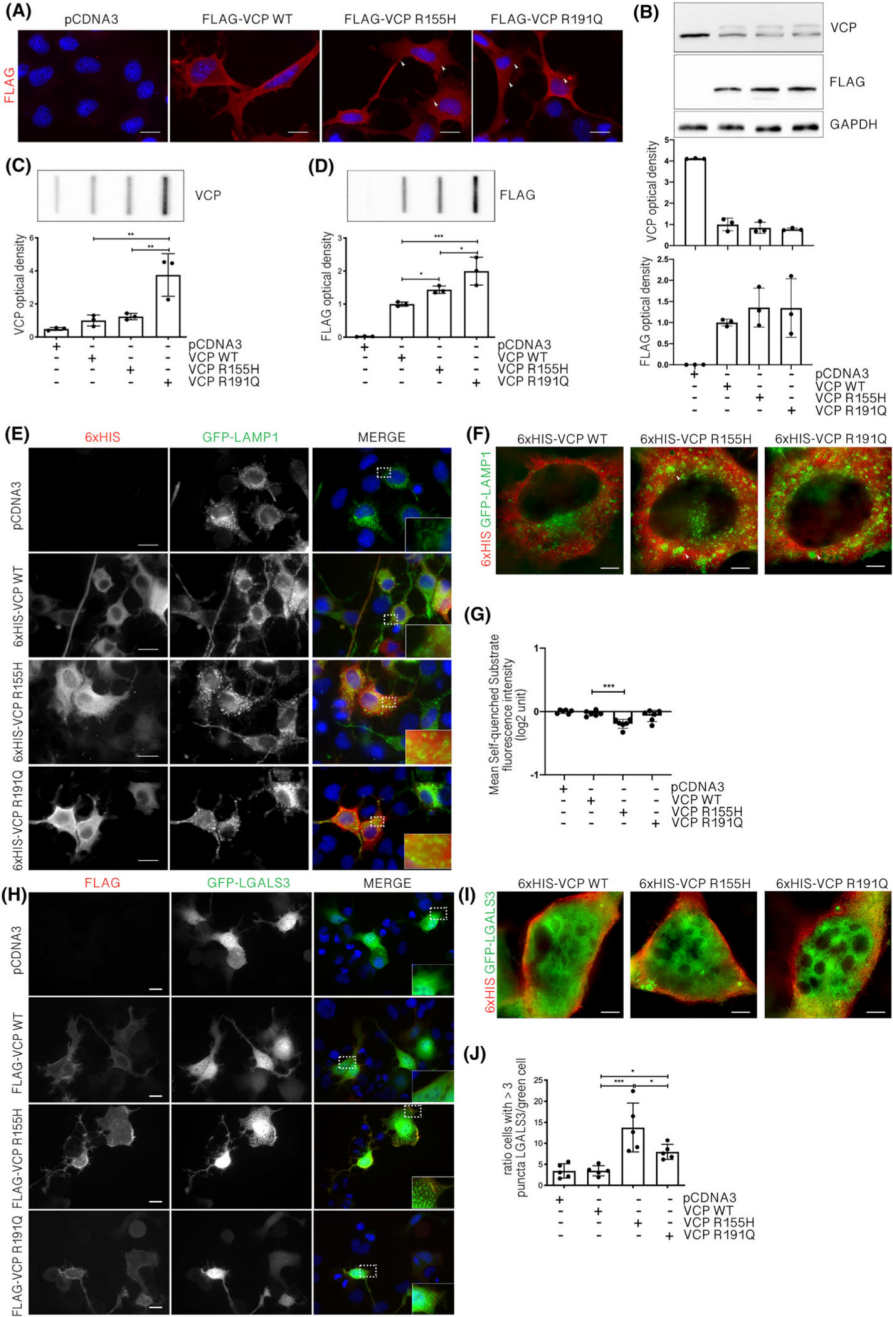
Valosin-containing protein (VCP) mutants aggregate and induce lysosomal damage in NSC-34. (A) Immunofluorescence (IF) microscopy analysis (63× magnification) on NSC-34 cells overexpressing wild type (WT) or mutants FLAG-VCPs. Nuclei were stained with 4′,6-diamidino-2-phenylindole (DAPI) (blue), whereas FLAG-VCP with the anti-FLAG antibody (red). Scale bar, 10 μm. (B) A representative western blotting (WB) analysis of phosphate-buffered saline (PBS) extracts of NSC-34 overexpressing WT or mutants FLAG-VCPs. To visualise total VCP, an anti-VCP antibody was used. To visualise exogenous FLAG-VCP, an anti-FLAG antibody was used. Glyceraldehyde-3-phosphate dehydrogenase (GAPDH) was used as loading control (upper inset). Optical densitometry quantification of VCP (middle inset) and FLAG-VCP (lower inset) in WB was computed over three independent biological samples for each condition (*n* = 3) SD (one-way analysis of variance [ANOVA] followed by Fisher’s least significant difference [LSD] test). (C,D) Filter trap assay (FTA) (upper inset) of PBS extracts of NSC-34 overexpressing WT or mutants FLAG-VCPs. To visualise total VCP, an anti-VCP antibody was used (C). To visualise exogenous FLAG-VCP, an anti-FLAG antibody was used (D). Bar graph represents FTA mean relative optical density computed over three independent biological samples for each condition (*n* = 3) SD (one-way ANOVA followed by Fisher’s LSD test; *p < 0.05, **p < 0.01, ***p < 0.001). (E) IF microscopy analysis (63× magnification) on NSC-34 cells overexpressing WT or mutants 6xHIS-VCPs and GFP-LAMP1. 6xHIS-VCPs were stained with an anti-6xHIS antibody (red), LAMP1 was visualised as GFP-LAMP1 (green) and nuclei were stained with DAPI (blue). A 2.5× magnification of selected areas is shown. Scale bar, 10 μm. (F) Stimulated emission depletion (STED) microscopy analysis on NSC-34 cells overexpressing WT or mutants 6xHIS-VCPs and GFP-LAMP1. 6xHIS-VCPs were stained with an anti-6xHIS antibody (red), and LAMP1 was visualised as GFP-LAMP1 (green). Scale bar, 5 μm. (G) Cytofluorimetric analysis performed on NSC-34 cells overexpressing WT or mutants FLAG-VCPs treated with Lysosome-Specific Self-Quenched Substrate. Mean fluorescence intensity was measured (*n* = 6) (one-way ANOVA followed by Fishers LSD test; ***p < 0.001). (H) IF microscopy analysis (40× magnification) on NSC-34 cells overexpressing WT or mutants FLAG-VCPs and GFP-LGALS3. FLAG-VCPs were stained with an anti-FLAG antibody (red), galectin-3 was visualised as GFP-LGALS3 (green) and nuclei were stained with DAPI (blue). A 2× magnification of selected areas is shown. Scale bar, 10 μm. (I) STED microscopy analysis on NSC-34 cells overexpressing WT or mutants 6xHIS-VCPs and GFP-LGALS3. 6xHIS tagged VCPs were detected using an anti-6xHIS antibody red and galectin-3 as GFP-LGALS3 (green). Scale bar, 5 μm. (J) The bar graph represents the quantification of the percentage of cells with >3 GFP-LGALS3 puncta after transfection with pCDNA3, WT or mutants FLAG-VCPs; the fields were randomly selected, and at least 100 cells for each sample were counted over 5 independent biological samples for each condition (*n* = 5) SD (one-way ANOVA followed by Fisher’s LSD test; *p < 0.05, ***p < 0.001)

We analysed how these acutely induced structures associate with altered lysosomal membrane conformation in response to VCP mutants. Because VCP mutants form insoluble species in skeletal muscles [20,55], we studied whether this aggregation-prone behaviour is maintained in motoneuronal cells. We overexpressed FLAG-tagged WT or mutant VCPs in motoneurons and found that both the VCP mutants are mostly diffused in the cytoplasm but that there are a few small cytoplasmic FLAG-positive puncta (evidenced by the arrows) of tiny protein aggregates (Figure 2A), not present in control cells. In WB, the anti-FLAG antibody recognised similar levels and turnover of the exogenous FLAG-tagged VCPs (Figure 2B). On the contrary, the anti-VCP antibody, which recognises both exogenous and endogenous VCP, showed that the exogenous expression of human VCP (either WT or mutated) reduced the endogenous murine VCP levels (Figure 2B). By FTA, we further characterised the aggregation propensity of VCP mutants (Figure 2C,D) using both the anti-VCP and the anti-FLAG antibodies. Exogenous VCPs formed high molecular weight PBS-insoluble species, which were particularly elevated for R191Q VCP. Thus, these data recapitulate those of IF analyses and correlate with published data obtained in muscle cells, demonstrating that both R155H VCP and R191Q VCP form aggregates in motoneuronal cells.

Generally, protein aggregates alter different cellular pathways and the dynamics of organelles, including lysosomes [56–59]. We thus studied by conventional and STED microscopy whether VCP mutants lead to alterations of lysosome dynamics, by monitoring the distribution of GFP-tagged LAMP1, a protein associated with lysosomal membranes. Notably, both VCP mutants, but not WT, induced lysosome enlargement (Figure 2E) with a filled lumen (Figure 2F, arrowheads). These morphological alterations confirmed the EM studies (Figure 1C), which also showed altered morphology and enlarged lysosomes. To determine whether aberrant lysosome structure and morphology correlate with altered lysosome function, we analysed the activity of lysosomal enzymes. We found a decrease in lysosomal enzyme activity in cells expressing R155H VCP, but not WT VCP (Figure 2G). The presence of LMP impairment was studied by analysing the distribution of GFP-tagged galectin-3 (GFP-LGALS3), a small sugar-binding protein that accumulates on the lysosomal membrane only upon its permeabilization. In basal condition, GFP-LGALS3 is present diffusely in the cytoplasm and no puncta can be observed (Figure 2H); however, when lysosomal damage and LMP are induced, GFP-LGALS3 translocates and binds to the leaky lysosomes, forming puncta on the damaged lysosomal membrane. We observed GFP-LGALS3 puncta in cells expressing VCP mutants (Figure 2H,I) but not WT VCP. The quantification of GFP-LGALS3 puncta (Figure 2J), based on a standardised procedure that considers positive cells as containing at least 3 GFP-LGALS3 puncta per cell [44], confirmed that both VCP mutants, particularly R155H VCP, significantly increased lysosomal damage compared with control VCP. Together, these results show for the first time that the acute expression of VCP mutants is sufficient to cause detrimental alterations in lysosomal stability and dynamics.

### WT VCP modulation decreases damaged lysosome levels

Upon pharmacological stress or exposure to misfolded proteins, lysophagy is activated to trigger the clearance of damaged lysosomes; however, VCP mutants may compromise this process [9]. We studied whether VCP mutants exerted a deleterious effect on damaged lysosomes in motoneurons. To study this issue, we chemically induced subtle lysosomal damage using trehalose, which differs from LLOMe, a known lysosomotropic agent. Trehalose induces limited, transient lysosomal damage that activates a protective autophagic response potentiating lysophagy [44]. In fact, we found that LLOMe treatment is cytotoxic to motoneuronal cells, whereas trehalose exposure for up to 18 h has no effect on cell viability (data not shown). Moreover, lysosomal damage induced by 1 h of LLOMe treatment is comparable with that of 6 h of trehalose treatment, but a major difference between the treatments is found in the recovery phase. Indeed, trehalose induces activation of autophagy, which allows cells to recover from the lysosomal damage, preventing cell death, whereas previous studies showed that LLOMe induces a decrease in cell viability because only 50% of cells recover after LLOMe treatment [44]. Thus, we could consider trehalose treatment as a disease model only at 6 h of treatment without considering the recovery that follows.

**FIGURE 3:**
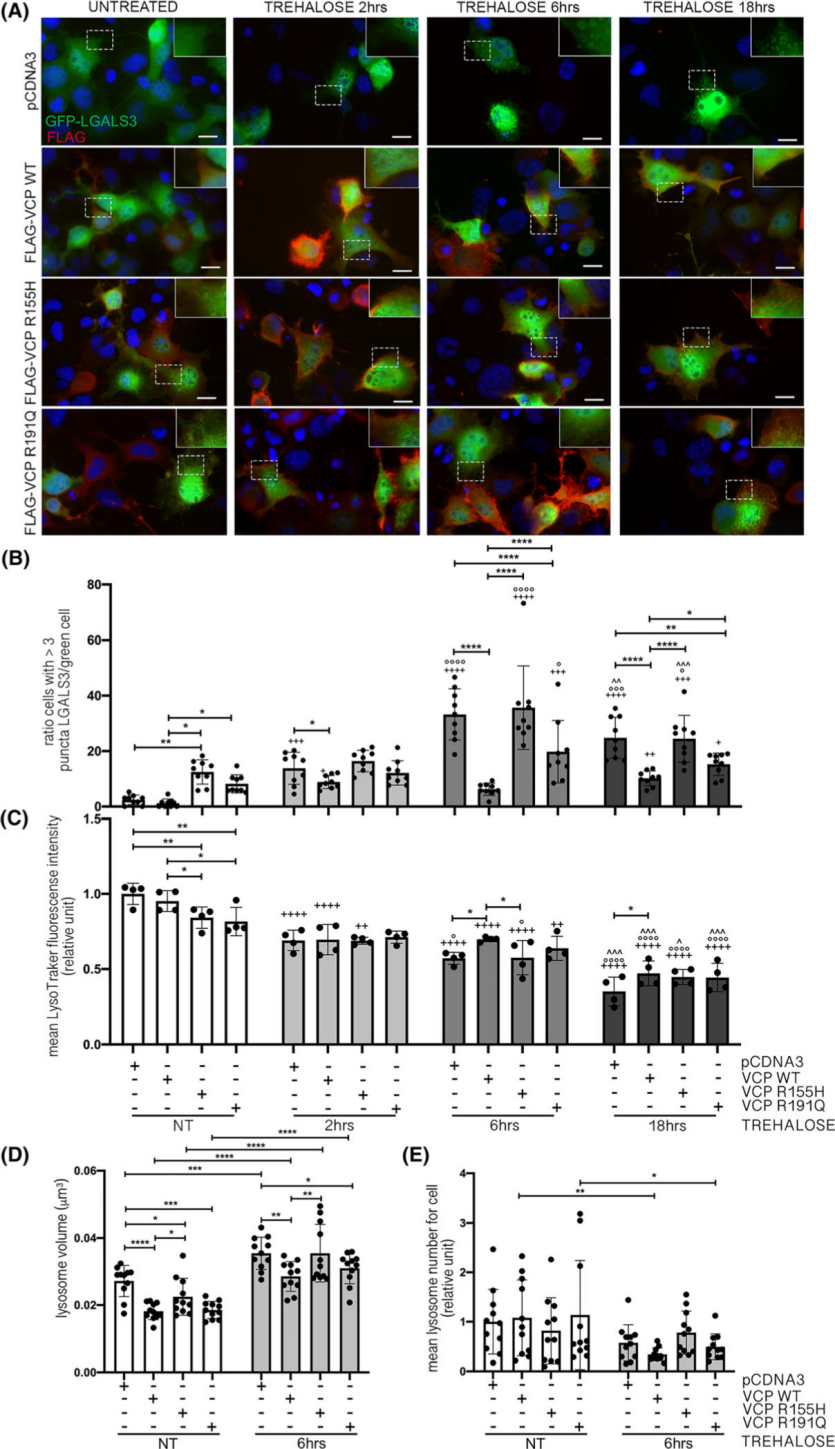
Wild type (WT) valosin-containing protein (VCP) modulation decrease levels of chemically damaged lysosomes. (A) Immunofluorescence (IF) microscopy analysis (40× magnification) on NSC-34 cells overexpressing WT or mutants FLAG-VCPs and GFP-LGALS3 and treated with trehalose for different time points. FLAG-VCPs were stained with anti-FLAG antibody (red), galectin-3 was visualised as GFP-LGALS3 (green) and nuclei were stained with 4′,6-diamidino-2-phenylindole (DAPI) (blue). A 2× magnification of selected areas is shown. Scale bar, 10 μm. (B) The bar graph represents the quantification of percentage of cells with >3 GFP-LGALS3 puncta after transfection with pCDNA3, WT or mutants FLAG-VCPs and after trehalose treatment at different time points; the fields were randomly selected, and at least 100 cells for each sample were counted over 9 independent biological samples for each condition (*n* = 9) SD (two-way analysis of variance [ANOVA] with Fisher’s least significant difference [LSD] test; *p < 0.05, **p < 0.01, ****p < 0.0001; ^+^*p* < 0.05, ^++^*p* < 0.01, ^+++^*p* < 0.001, ^++++^*p* < 0.0001, vs NT; °*p* < 0.05, °°°*p* < 0.001, °°°°*p* < 0.0001, vs 2-h trehalose; ^^*p* < 0.01, ^^^*p* < 0.001, vs 6h trehalose). (C) The bar graph represents the quantification of the mean LysoTracker fluorescence intensity cytofluorimetric analysis performed on NSC-34 cells overexpressing WT or mutants FLAG-VCPs, treated with trehalose for different time periods and labelled with LysoTracker Green. Mean fluorescence intensity was measured (*n* = 4) (two-way ANOVA with Fisher’s LSD test; *p < 0.05, **p < 0.01; ^++^*p* < 0.01, ^++++^*p* < 0.0001, vs NT; °*p* < 0.05, °°°°*p* < 0.0001, vs 2-h trehalose; ^*p* < 0.05, ^^^*p* < 0.001, vs 6-h trehalose). (D) The bar graph represents the quantification of lysosomes volume of NSC-34 after transfection with pCDNA3, WT or mutants FLAG-VCPs and after 6-h trehalose treatment. The fields were randomly selected, and at least 50 cells for each sample were counted over 11 independent biological samples for each condition (*n* = 11) SD (two-way ANOVA with Fisher’s LSD test; *p < 0.05, **p < 0.01, ***p < 0.001, ****p < 0.0001). (E) The bar graph represents the number of lysosomes of NSC-34 after transfection with pCDNA3, WT or mutants FLAG-VCPs and after 6-h trehalose treatment. The fields were randomly selected, and at least 50 cells for each sample were counted over 11 independent biological samples for each condition (*n* = 11) SD (two-way ANOVA with Fisher’s LSD test; *p < 0.05, **p < 0.01)

Here, we found that in control cells, trehalose increased lysosomal damage with a peak at 6 h of treatment. Trehalose-induced damage was reversed by WT VCP overexpression (Figure 3A,B). On the contrary, the two VCP mutants exerted different effects: trehalose-treated cells expressing R155H VCP showed a level of lysosomal damage comparable with that observed in control cells at all time points analysed (Figure 3B); whereas trehalose-treated cells expressing R191Q VCP displayed lower levels of lysosomal damage compared with control cells but higher levels compared with cells overexpressing WT VCP. We further analysed lysosome functionality in these conditions by evaluating lysosomal acidification (Figure 3C), using LysoTracker^®^, a compound highly selective for acidic organelles like lysosomes, in which its fluorescence is activated. Thus, by comparing each condition with the control, the fluorescence intensity allows evaluation of any alteration of lysosome acidification.

We found that in untreated conditions, VCP mutants decrease lysosomal acidification. A progressive decrease in acidification was also triggered by trehalose treatment, which was found to be reversed at 6 and 18 h by the overexpression of the WT VCP but not of the VCP mutants. Finally, we quantified lysosomal size and number at 6 h of trehalose treatment, when lysosomal damage was most visible, and compared it with untreated conditions (Figure 3D,E). We found that trehalose treatment increased lysosomal size. This increase was prevented by the overexpression of WT VCP and only partially prevented by R191Q VCP, whereas R155H VCP was inactive. Moreover, we detected a trend for decreased lysosomal number in the presence of trehalose treatment, but the levels did not show any significant difference in the presence of WT VCP or mutants.

**FIGURE 4:**
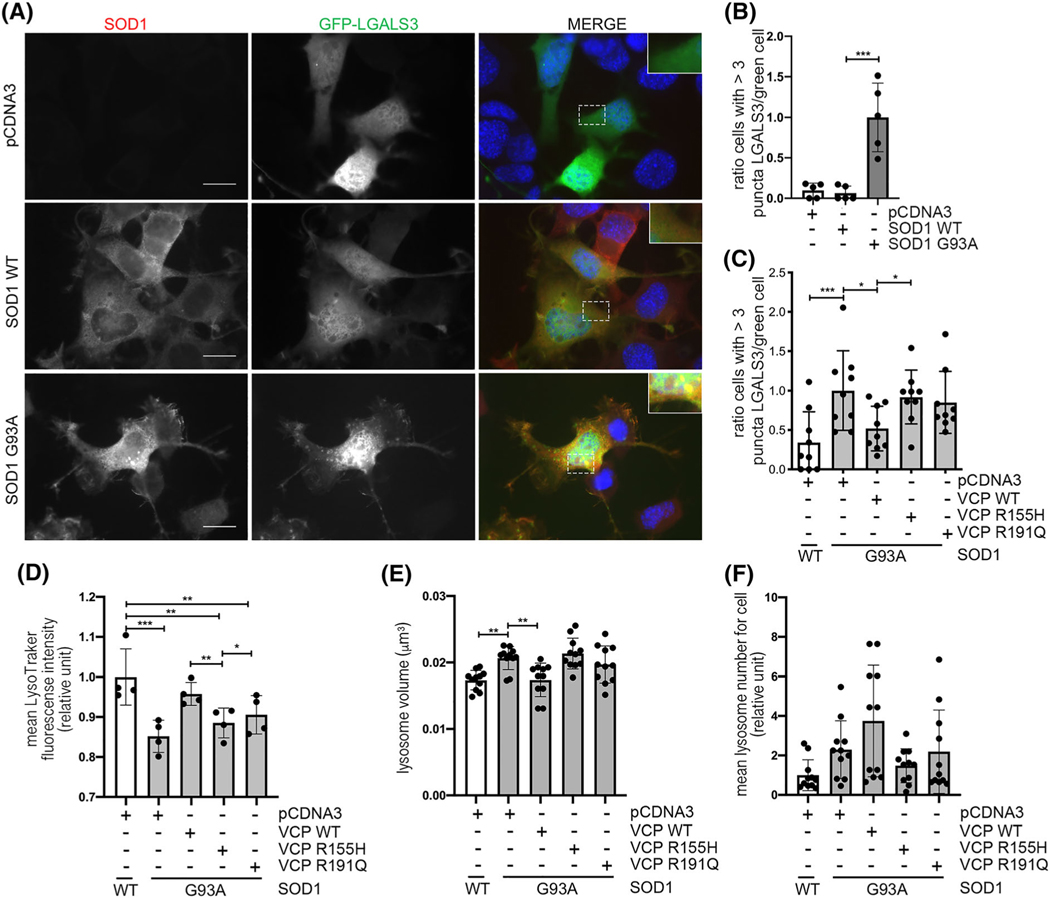
Wild type (WT) valosin-containing protein (VCP) modulation decreases levels of lysosomal damage induced by SOD1 mutant. (A) Immunofluorescence (IF) microscopy analysis (63× magnification) on NSC-34 cells overexpressing WT SOD1 or G93A SOD1 and GFP-LGALS3. SOD1 was stained with an anti-SOD1 antibody (red), galectin-3 was visualised as GFP-LGALS3 (green) and nuclei were stained with 4′,6-diamidino-2-phenylindole (DAPI) (blue). A 2× magnification of selected areas is shown. Scale bar, 10 μm. (B) The bar graph represents the quantification of percentage of cells with >3 GFP-LGALS3 puncta after transfection with pCDNA3, WT SOD1 or G93A SOD1; the fields were randomly selected and at least 100 cells for each sample were counted over 5 independent biological samples for each condition (*n* = 5) ±SD (Unpaired *t* test with Welch’s correction; **p < 0.001) (C) The bar graph represents the quantification of percentage of cells with >3 GFP-LGALS3 puncta after transfection with pCDNA3, WT or mutants FLAG-VCPs and WT SOD1 or G93A SOD1; the fields were randomly selected, and at least 100 cells for each sample were counted over 9 independent biological samples for each condition (*n* = 9) ± SD (one-way analysis of variance [ANOVA] followed by Fisher’s least significant difference [LSD] test; *p < 0.05, ***p < 0.001). (D) The bar graph represents the quantification of the mean LysoTracker fluorescence intensity cytofluorimetric analysis performed on NSC-34 transfected with pCDNA3, WT or mutants FLAG-VCPs and WT SOD1 or G93A SOD1 and labelled with LysoTracker Green. Mean fluorescence intensity was measured (*n* = 4) (One-way ANOVA with Fisher’s LSD test; *p < 0.05, **p < 0.01, ***p < 0.001). (E) The bar graph represents the quantification of lysosome volume of NSC-34 transfected with pCDNA3, WT or mutants FLAG-VCPs and WT SOD1 or G93A SOD1. The fields were randomly selected, and at least 50 cells for each sample were counted over 11 independent biological samples for each condition (*n* = 11) ±SD (one-way ANOVA with Fisher’s LSD test; **p < 0.01) (F) The bar graph represents the number of lysosomes of NSC-34 after transfection pCDNA3, WT or mutants FLAG-VCPs and WT SOD1 or G93A SOD1. The fields were randomly selected, and at least 50 cells for each sample were counted over 11 independent biological samples for each condition (*n* = 11) ±SD (one-way ANOVA with Fisher’s LSD test)

Lysosomal damage is also triggered by overexpression of some misfolded proteins, including the mutant forms of the antioxidant enzyme SOD1, which is responsible for fALS (Figure 4A,B). Therefore, we analysed the effect of WT VCP and mutants on damaged lysosomes generated upon exposure to misfolded mutant G93A SOD1 (using WT SOD1 as control). Overexpression of WT VCP fully rescued lysosomal damage induced by G93A SOD1 (Figure 4C). Conversely, the VCP mutants were unable to counteract G93A SOD1-induced lysosomal damage, as monitored by counting the number of GFP-LGALS3 puncta (Figure 4C). We then evaluated lysosomal acidification, size and number under these conditions (Figure 4D–F). G93A SOD1 decreased lysosome acidification and increased lysosomal size if compared with cells expressing WT SOD1. This was reversed by the overexpression of WT VCP but not by the VCP mutants. As with trehalose treatment, we could not detect a significant difference in the number of lysosomes.

Together, these data indicate that enhancing WT VCP activity rescues lysosomal damage induced either by trehalose or by G93A SOD1 expression. In contrast, this protective activity of VCP is partly compromised for the disease-linked R155H and R191Q VCP mutants. The data obtained in motoneurons are in line with those collected so far in muscle [37], strongly supporting the notion that similar loss-of-function mechanisms take place in VCP associated MSP and in ALS.

### VCP mutants activate autophagy

**FIGURE 5:**
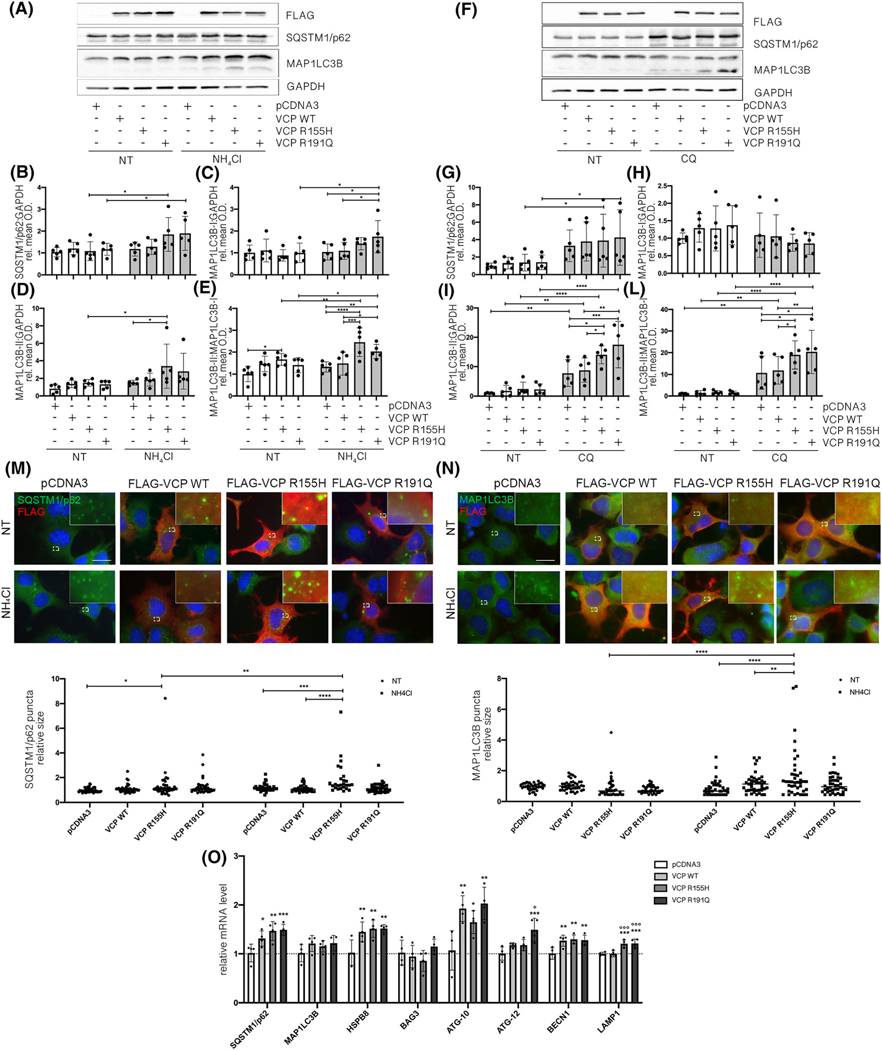
Valosin-containing protein (VCP) mutants activate autophagy. (A) A representative western blotting (WB) of phosphate-buffered saline (PBS) extracts of NSC-34 overexpressing wild type (WT) or mutants FLAG-VCPs and treated with NH_4_Cl. To visualise FLAG-VCP, an anti-FLAG antibody was used. To visualise the autophagic markers MAP 1LC3B and SQSTM1/p62, an anti-MAP 1LC3B antibody and an anti-SQSTM1/p62 antibody were used respectively. Glyceraldehyde-3-phosphate dehydrogenase (GAPDH) was used as loading control. (B–E) Quantification of WB analysis presented in Figure A. (B) The bar graph represents the mean relative optical density quantification of SQSTM1/ p62 detected by WB using an anti-SQSTM1/p62 antibody. (C) The bar graph represents the mean relative optical density quantification of MAP 1LC3B-I detected by WB (upper lane considered) using an anti-MAP1LC3B antibody. (D) The bar graph represents the mean relative optical density quantification of MAP1LC3B-II detected by WB (lower lane considered) using an anti-MAP1LC3B antibody. (E) The bar graph represents the ratio of the mean relative optical density quantification of MAP1LC3B-II detected by WB (lower lane considered) and of the mean relative optical density quantification of MAP1LC3B-I detected by WB (upper lane) using an anti-MAP1LC3B antibody. All quantification histograms were calculated using the mean SD for *n* = 5 independent replicates. (Two-way analysis of variance [ANOVA] with Fisher’s least significant difference [LSD] test; *p < 0.05, **p < 0.01, ***p < 0.001, ****p < 0.0001). (F) A representative WB of PBS extracts of NSC-34 overexpressing WT or mutants FLAG-VCPs and treated with CQ. (G–L) Quantification of WB analysis presented in Figure F. (G) The bar graph represents the mean relative optical density quantification of SQSTM1/p62 detected by WB using an anti-SQSTM1/p62 antibody. (H) The bar graph represents the mean relative optical density quantification of MAP1LC3B-I detected by WB (upper lane considered) using an anti-MAP1LC3B antibody. (I) The bar graph represents the mean relative optical density quantification of MAP1LC3B-II detected by WB (lower lane considered) using an anti-MAP 1LC3B antibody. (L) The bar graph represents the ratio of the mean relative optical density quantification of MAP1LC3B-II detected by WB (lower lane considered) and of the mean relative optical density quantification of MAP1LC3B-I detected by WB (upper lane) using an anti-MAP1LC3B antibody. All quantification histograms were calculated using the mean SD for *n* = 5 independent replicates. (Two-way ANOVA with Fisher’s LSD test; *p < 0.05, **p < 0.01, ***p < 0.001, ****p < 0.0001). (M) Immunofluorescence (IF) microscopy analysis (63× magnification) of NSC-34 overexpressing WT or mutants FLAG-VCPs (upper inset). FLAG-VCP was stained with an anti-FLAG antibody (red), SQSTM1/p62 was stained with an anti-SQSTM1/p62 antibody (green) and nuclei were stained with DAPI (blue). An 8× magnification of selected areas is shown. Scale bar, 10 μm. The bar graph (lower inset) represents quantification of SQSTM1/p62 puncta average size per cell; 40 cells were analysed for each condition (two-way ANOVA with Dunnetts test and Šidáks test *p < 0.05, **p < 0.01, ***p < 0.001, ****p < 0.0001). (N) IF microscopy analysis (63× magnification) of NSC-34 overexpressing WT or mutants FLAG-VCPs (upper inset). FLAG-VCP was stained with an anti-FLAG antibody (red), MAP 1LC3B was stained with an anti-MAP1LC3B antibody (green) and nuclei were stained with DAPI (blue). An 8× magnification of selected areas is shown. Scale bar, 10 μm. The bar graph (lower inset) represents quantification of MAP1LC3B puncta average size per cell; 40 cells were analysed for each condition (two-way ANOVA with Dunnett’s test and Šidák’s test; **p < 0.01, ****p < 0.0001). (O) Real-time quantitative polymerase chain reaction (RT-qPCR) for SQSTM1/p62, MAP1LC3B, HSPB8, BAG3, ATG-10, ATG-12, BECN1 and LAMP1 mRNA normalised with Rplp0 mRNA levels. Data are means ± SD of 4 independent samples (one-way ANOVA with Fisher’s LSD test; *p < 0.05, **p < 0.01, ***p < 0.001 vs pCDNA3; °*p* < 0.05, °°°*p* < 0.001 vs WT VCP)

As previously mentioned, several pieces of data, including ours, show that damaged lysosomes activate autophagy, which promotes their clearance [9,44,60]. We demonstrated that VCP mutants acutely induce LMP and, unlike WT VCP, fail to rescue from lysosomal damage. Thus, we analysed whether lysosomal damage induced by VCP mutants is capable of enhancing the autophagic flux in motoneurons. We evaluated both SQSTM1/p62 accumulation and MAP1LC3B-I conversion to MAP1LC3B-II, the lipidated and activated form, in motoneurons overexpressing WT or mutant VCP (Figure 5A–L). We performed the analyses either in basal conditions or upon NH_4_Cl or CQ treatment that prevents autophagolysosomal degradation of both SQSTM1/p62 and MAP1LC3B-II [61]. We found increased SQSTM1/p62 levels when the autophagic flux was blocked by NH_4_Cl in the presence of VCP mutants (Figure 5B). In the same conditions, we found a moderate increase in MAP1LC3B-I levels in the presence of R191Q VCP compared with control conditions (Figure 5C). By contrast, the levels of MAP1LC3B-II were increased by R155H VCP overexpression (Figure 5D). The MAP1LC3B-II/MAP1LC3B-I ratio clearly showed that both VCP mutants are able to enhance the autophagy response in motoneurons (Figure 5E). These data were confirmed and even strengthened when we used CQ to block the autophagic flux, because CQ is a stronger inhibitor of autophagy. SQSTM1/p62 and MAP1LC3B-II levels increased in the presence of both VCP mutants (Figure 5H,I). In addition, MAP1LC3B-II/MAP1LC3B-I ratio confirmed that both mutants promote autophagic flux (Figure 5I).

The levels of the monomeric soluble autophagy receptor SQSTM1/p62 were only partially increased by VCP mutants (Figure 5B), but its subcellular distribution was completely modified. In particular, R155H VCP increased the size of SQSTM1/p62 puncta (also known as SQSTM1/p62-bodies) (Figure 5M). This relocalisation of SQSTM1/p62 into SQSTM1/p62-bodies is not necessarily combined with modification of the total protein levels, but it is a clear sign of activation of autophagic flux [62]. Furthermore, in line with the WB data, IF analysis (Figure 5N) showed an increase in the size of MAP1LC3B puncta in the presence of VCP mutants upon NH_4_Cl treatment, confirming the MAP1LC3B conversion correlated with autophagy activation. Finally, by analysing the expression of autophagy-related genes, we found that the increase of autophagic flux was associated with autophagic gene upregulation (Figure 5O). Indeed, WT and mutant VCP promoted the expression of *SQSTM1*, *HSPB8*, *ATG-10* and *BECN1* genes; *ATG-12* was specifically upregulated in presence of R191Q VCP; *LAMP1* expression increased in presence of both VCP mutants. Unexpectedly, neither WT VCP nor mutants promoted *BAG3* expression. Together, these data show that VCP mutants (R155H and R191Q) selected for this study enhance the autophagic flux.

### VCP mutants specifically increase TFE3 nuclear levels

Once determined that both VCP mutants enhance the autophagic flux and increase the expression of some autophagy-related genes, we dissected out the pathway mediating this activity. We found that clearance of damaged lysosomes depends on transcription factors regulating the Coordinated Lysosomal Expression and Regulation (CLEAR) genes [63,64]. The main CLEAR gene regulators are TFEB, a master regulator of autophagy, and TFE3.

**FIGURE 6:**
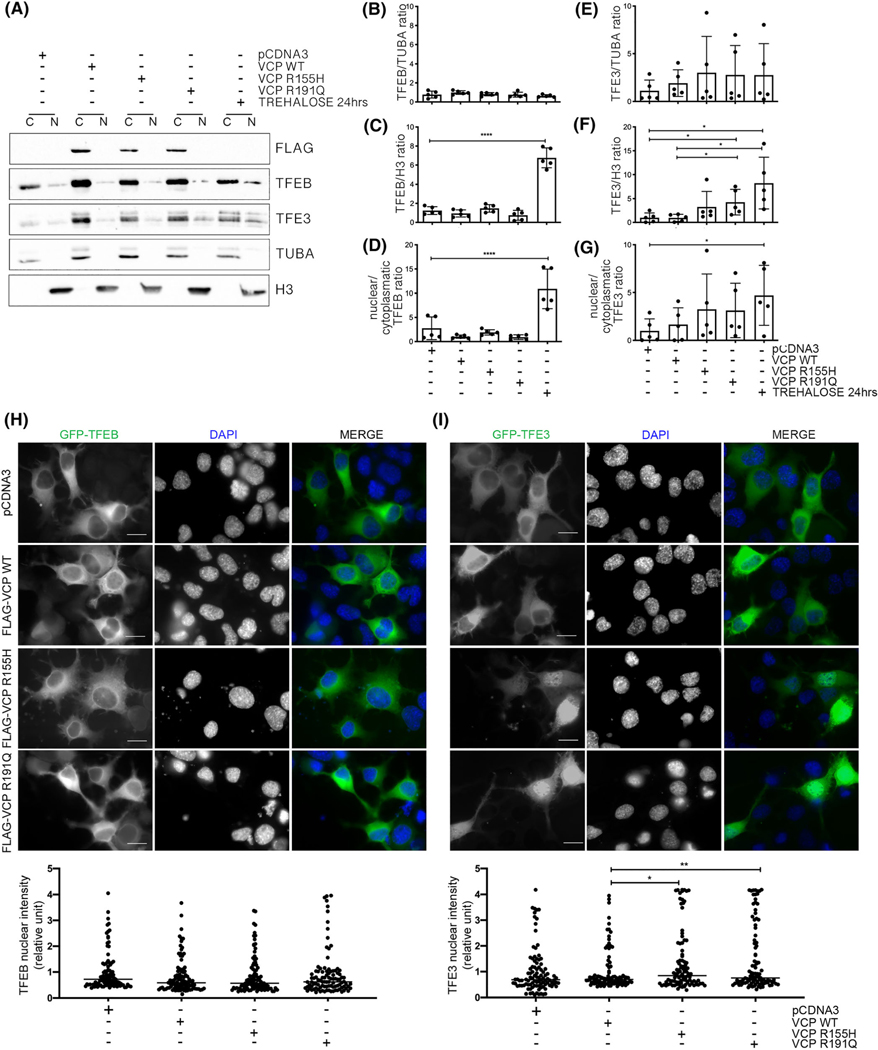
Valosin-containing protein (VCP) mutants specifically increase TFE3 nuclear levels. (A) A representative western blotting (WB) analysis of cytoplasmic (C) and nuclear (N) extracts of NSC-34 overexpressing wild type (WT) or mutants FLAG-VCPs. To visualise FLAG-VCP, an anti-FLAG antibody was used. To visualise transcription factors transcription factor EB (TFEB) and TFE3, an anti-TFEB antibody and an anti-TFE3 antibody were used, respectively. α-Tubulin (TUBA) was used as loading control of the cytoplasmic fraction. Histone 3 (H3) was used as loading control of the nuclear fraction. (B–G) Quantification of WB analysis presented in Figure A. (B) The bar graph represents the mean relative optical density quantification of cytoplasmic fraction of TFEB detected by WB using an anti-TFEB antibody. (C) The bar graph represents mean relative optical density quantification of nuclear TFEB detected by WB using an anti-TFEB antibody. (D) The bar graph represents the ratio of the mean relative optical density quantification of nuclear TFEB and the mean relative optical density quantification of cytoplasmic TFEB detected by WB using an anti-TFEB antibody. (E) The bar graph represents mean relative optical density quantification of cytoplasmic fraction of TFE3 detected by WB using an anti-TFE3 antibody. (F) The bar graph represents mean relative optical density quantification of nuclear TFE3 detected by WB using an anti-TFE3 antibody. (G) The bar graph represents the ratio of the mean relative optical density quantification of nuclear TFE3 and of the mean relative optical density quantification of cytoplasmic TFE3 detected by WB using an anti-TFE3 antibody. All quantification histograms were calculated using the mean ± SD for 5 independent samples (one-way analysis of variance [ANOVA] followed by Fisher’s least significant difference [LSD] test and the unpaired *t* test with Welch’s correction; *p < 0.05, **p < 0.01). (H) Upper inset shows fluorescence microscopy analysis (63× magnification) of NSC-34 overexpressing WT or mutants FLAG-VCPs and GFP-TFEB. TFEB was visualised as GFP-TFEB (green), and nuclei were stained with DAPI (blue). Scale bar, 10 μm. The bar graph (lower inset) represents the quantification of TFEB nuclear intensity; the fields were randomly selected, and at least 100 cells were analysed for each condition (one-way ANOVA with Fishers LSD test). (I) Upper inset shows fluorescence microscopy analysis (63× magnification) of NSC-34 overexpressing WT or mutants FLAG-VCPs and GFP-TFE3. TFE3 was visualised as GFP-TFE3 (green) and nuclei were stained with DAPI (blue). Scale bar, 10 μm. The bar graph (lower inset) represents the quantification of TFE3 nuclear intensity; the fields were randomly selected, and at least 100 cells were analysed for each condition (one-way ANOVA with Fisher’s LSD test; *p < 0.05, **p < 0.01).

We thus analysed whether VCP mutants induce TFEB and TFE3 activation. Both transcription factors migrate into the nucleus only when activated. By nuclear/cytoplasmic fractionation studies, we found that while trehalose treatment induced TFEB nuclear localisation, the acute overexpression of VCP mutants did not alter TFEB localisation (Figure 6A–D). Conversely, we found that nuclear TFE3 localisation was enhanced by the expression of both VCP mutants, especially R191Q VCP (Figure 6A,E–G). By IF, we confirmed the nuclear TFE3 localisation induced by VCP mutants (Figure 6I). As for the fractionation studies, TFEB remained confined to the cytoplasm in all VCP conditions tested (Figure 6H). Conversely, TFE3 migrated into the nucleus in the presence of both VCP mutants (Figure 6I). These data differ from those of Arhzaouy et al. [37], who found that VCP mutant expression led to stabilisation of TFEB activation, but this occurs only in very old VCP mice, while we have proved that the expression of mutant VCP is not sufficient to acutely trigger the TFEB pathway.

### PPP3CB mediates TFE3 translocation induced by VCP mutants

**FIGURE 7:**
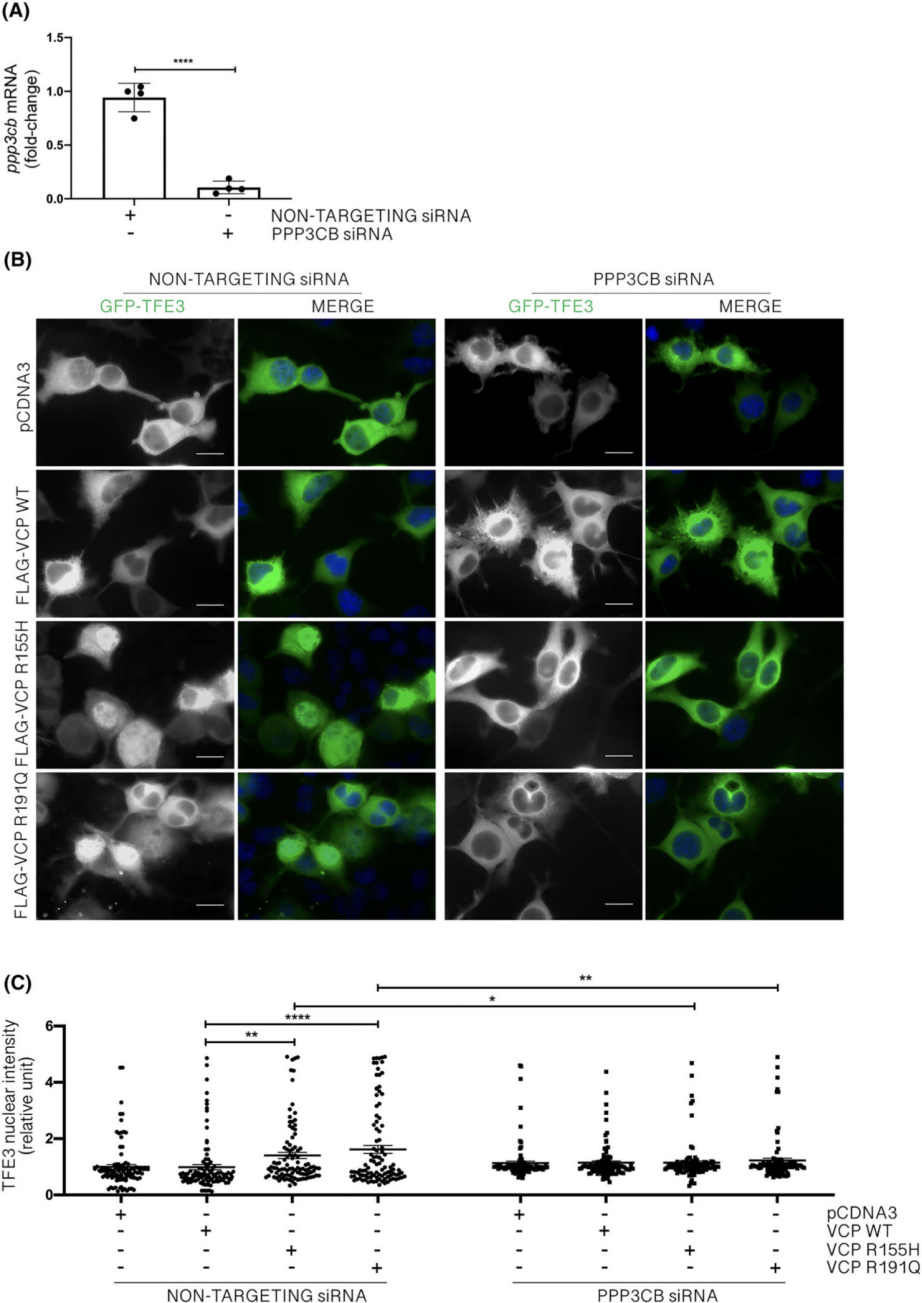
PPP3CB mediates TFE3 translocation induced by valosin-containing protein (VCP) mutants. (A) Real-time quantitative polymerase chain reaction (RT-qPCR) for PPP3CB mRNA normalised with Rplp0 mRNA levels. Data are means ± SD of 4 independent samples (unpaired student *t* test; ****p < 0.0001). (B) Fluorescence microscopy analysis (63× magnification) of NSC-34 overexpressing wild type (WT) or mutants FLAG-VCPs and transfected with PPP3CB or non-targeting siRNAs. TFE3 was visualised as GFP-TFE3 (green), and nuclei were stained with 4′,6-diamidino-2-phenylindole (DAPI) (blue). Scale bar, 10 μm. (C) The bar graph represents the quantification of TFE3 nuclear intensity; the fields were randomly selected, and at least 100 cells were analysed for each condition (two-way analysis of variance [ANOVA] with Fisher’s least significant difference [LSD] test; *p < 0.05, **p < 0.01, ****p < 0.0001)

TFE3 nuclear translocation and activation are triggered by its dephosphorylation by the Ca^2+^-dependent phosphatase PPP3CB [65]. Thus, we silenced PPP3CB (Figure 7A) in cells overexpressing WT or mutant VCP (Figure 7B,C). We found that whereas non-targeting siRNA cells showed increased GFP-TFE3 nuclear levels in the presence of VCP mutants, PPP3CB siRNA abolished TFE3 nuclear translocation induced by VCP mutants. Thus, TFE3 activation and nuclear translocation induced by VCP mutants depend on PPP3CB. This suggests that TFE3, but not TFEB, mediates the response to LMP possibly via lysosomal Ca^2+^ release in the cytoplasm, which activates PPP3CB to promote TFE3 dephosphorylation and activation.

### WT VCP and mutants enhance G93A SOD1 clearance

**FIGURE 8:**
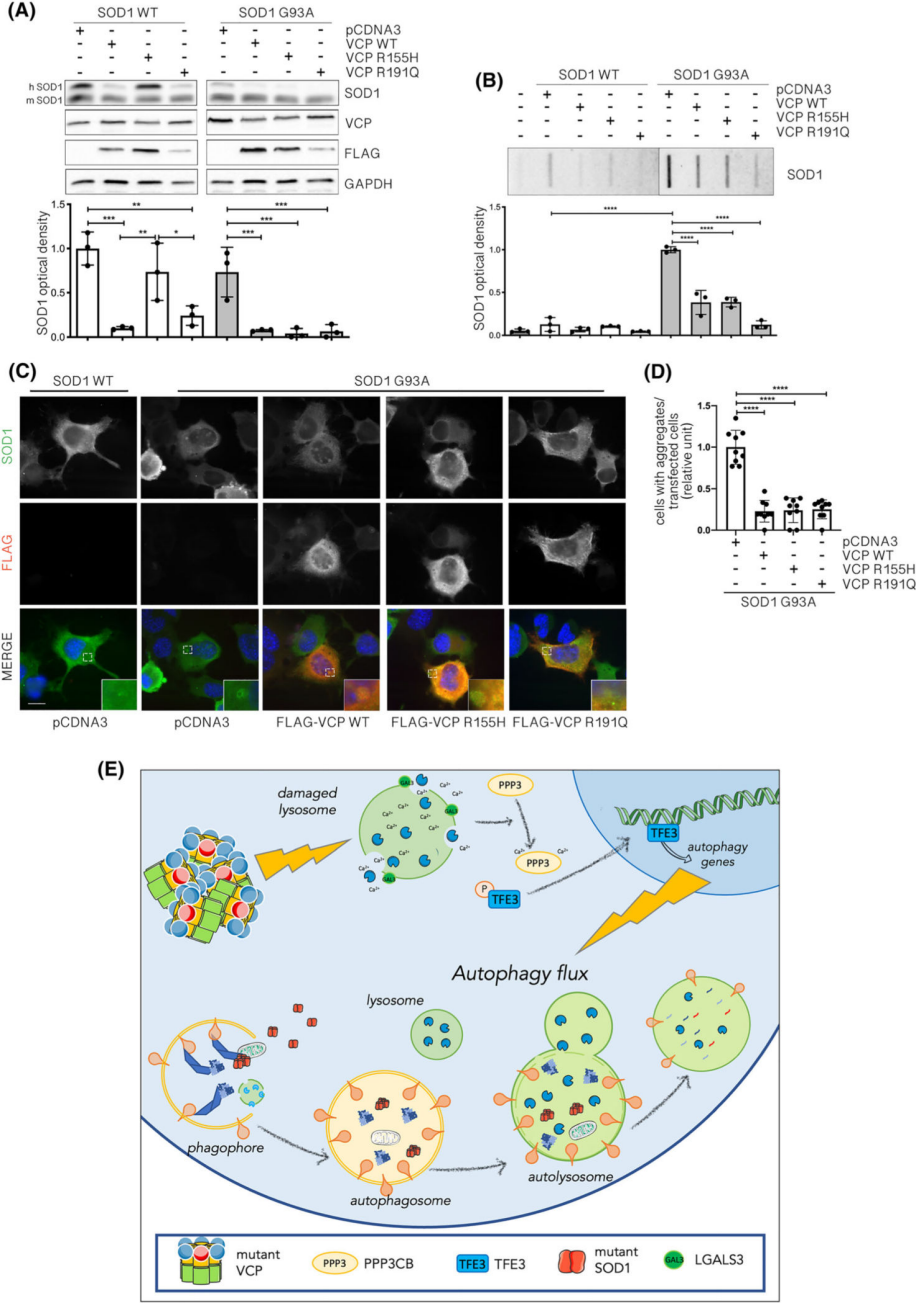
Wild type (WT) and mutants valosin-containing proteins (VCPs) enhance G93A SOD1 clearance. (A) A representative western blotting (WB) (upper inset) of phosphate-buffered saline (PBS) extracts added with SDS, from NSC-34 cells overexpressing WT SOD1 or G93A SOD1 and WT or mutants FLAG-VCPs. To visualise exogenous human (upper lane) or endogenous murine (lower lane) SOD1, an anti-SOD1 antibody was used. To visualise total VCP, an anti-VCP antibody was used. To visualise overexpressed FLAG-VCPs, an anti-FLAG antibody was used. Glyceraldehyde-3-phosphate dehydrogenase (GAPDH) was used as loading control. The bar graph represents the optical densitometry quantification of SOD1 detected in WB (lower inset) (one-way analysis of variance [ANOVA] with Fisher’s least significant difference [LSD] test; *p < 0.05, **p < 0.01, ***p < 0.001). (B) Filter trap assay (FTA) (upper inset) of PBS extracts from NSC-34 cells overexpressing WT or G93A SOD1 and WT or mutants FLAG-VCPs. To visualise SOD1, an anti-SOD1 antibody was used. The bar graph represents the mean relative optical density quantification of FTA (lower inset) (one-way ANOVA with Fisher’s LSD test; ****p < 0.0001). (C) Immunofluorescence (IF) microscopy analysis (63× magnification) on NSC-34 overexpressing WT SOD1 or G93A SOD1 and WT or mutants FLAG-VCPs. SOD1 was stained with an anti-SOD1 antibody (green), FLAG-VCPs were stained with an anti-FLAG antibody (red) and nuclei were stained with DAPI (blue). A 4× magnification of selected areas is shown. Scale bar, 10 μm. (D) The bar graph represents the quantification of cells with aggregates/transfected cells ratio of NSC-34 transfected WT SOD1 or G93A SOD1 and WT or mutants FLAG-VCPs; the fields were randomly selected, and at least 50 cells for each sample were counted over 9 independent biological samples for each condition (*n* = 9) ± SD (one-way ANOVA followed by Fisher’s LSD test; ****p < 0.0001). (E) Proposed model for the pathological mechanism exerted by VCP mutants in neuronal cells. VCP mutants aggregate and cause lysosomal size and morphology alteration and damage. Lysosomal damage, via PPP3CB, specifically causes TFE3 dephosphorylation and nuclear translocation. Active TFE3 promotes autophagy activation, which results in an increased flux compared with basal conditions. The increased autophagic flux leads to the clearance of damaged lysosomes via lysophagy and eventually enhances the clearance of misfolded proteins (e.g., mutant SOD1). This figure was created using Servier Medical Art templates, which are licenced under a Creative Commons Attribution 3.0 Unported Licence; https://smart.servier.com

Because VCP mutant overexpression leads to autophagy activation, we studied if this could trigger the clearance of misfolded aggregated proteins. WT and mutant VCP were thus co-expressed with human G93A SOD1, which is unstable, misfolds and forms insoluble aggregates associated with fALS [66–68]. As shown in Figure 8A, we found that the total SDS-soluble fraction of exogenous mutant human G93A SOD1 (upper band) was reduced by both WT and mutant VCP, without influencing the endogenous WT murine SOD1 (lower band). Also, the total SDS-soluble fraction of human WT SOD1 was decreased by WT VCP and R191Q VCP and totally cleared by R155H VCP (Figure 8A). Thus, WT VCP and mutants specifically clear the soluble forms of human SOD1. With the use of the FTA assay, we tested if WT VCP and mutants remove G93A SOD1 aggregates (Figure 8B). It is known that the PBS-insoluble G93A SOD1 levels are significantly higher than those of WT SOD1. We found that this insoluble fraction was decreased by both WT and mutant VCP in NSC-34 cells. IF analysis confirmed the decrease of G93A SOD1 aggregates in presence of VCP either WT or mutants (Figure 8D). In addition, in this condition, we observed that in the small fraction of cells containing aggregates, VCP is sequestered in the G93A SOD1 aggregates suggesting a reduction of the VCP bioavailability (and activity) in these cells (Figure 8C). Thus, WT VCP enhances the clearance of G93A SOD1 aggregates. Also, VCP mutants retain this capability in assisting misfolded protein aggregate degradation. Therefore, we suggest VCP mutant’s’ capability to clear away misfolded protein aggregates could be correlated with autophagy activation triggered by mutant VCP induced-lysosomal damage and enhanced-autophagic flux.

## DISCUSSION

MSP or IBMPFD, FTD and ALS are neurodegenerative/neuromuscular diseases characterised by proteostasis alterations. The most common familial forms of MSPs are associated with *VCP* mutations and the formation of ubiquitin- and TARDBP/TDP-43-positive inclusions, which are both clear signs of altered PQC [21]. The PQC system is modulated by VCP and assists the removal of misfolded aggregated proteins and of altered organelles, like damaged lysosomes [9]. Lysosomal damage from ruptured membranes leads to cell toxicity and death [69,70], and cells must activate their repair or degradation by lysophagy.

Here, we investigated possible pathological mechanisms for disease-associated *VCP* mutations. We found that VCP mutants accumulate into insoluble species in motoneuronal cells, and this gain of function alters cellular homeostasis. Indeed, VCP mutants also induce alterations in lysosomal size, morphology and enzyme activity in motoneurons. These alterations partially resemble those occurring on a subset of tubular lysosomes in muscle expressing VCP mutants [71,72]. We also proved that in motoneurons VCP mutants trigger lysosomal damage. Notably, WT VCP accelerates the clearance of lysosomes damaged either by specific drugs or by misfolded proteins, as demonstrated by other authors using more detrimental pharmacological approaches [9,37]. Here, we demonstrated for the first time in a motoneuronal model that this protective activity of WT VCP is lost in VCP mutants, suggesting a loss of function mechanism. Thus, a dual activity co-exists for VCP mutants on lysosomal dynamics that relies on both a gain and a loss of function mechanism. The loss of VCP activity may result in defects in removing ubiquitinated proteins from damaged lysosomes, as described by Papadopoulos et al. [9] or in promoting autophagy induction, as shown by Hill et al. [36]. These works suggest that lysosomal damage stabilisation exerted by VCP mutants could also be due to their loss of function in autophagy regulation. In any case, the presence of VCP mutants alters autophagy and lysophagy at different steps.

We found that VCP mutants positively modulate the autophagic flux, because R155H VCP increased the MAP1LC3B-I to MAP1LC3B-II conversion. MAP1LC3B-II increase is accompanied by a trend of SQSTM1/p62 accumulation into SQSTM1/p62-bodies and an upregulation of several autophagy-related genes. In addition, we demonstrated that WT VCP modulation in motoneurons leads to an increased clearance of mutant SOD1 aggregates, and this activity is retained by VCP mutants, possibly via an autophagic flux enhancement.

A novel aspect that emerged from our study is that TFE3, but not TFEB, mediates the rescue from VCP mutant-induced lysosomal damage. Lysosomal damage is known to cause activation of the CLEAR network through both TFEB and TFE3. Notably, we found that VCP mutants specifically promote TFE3 but not TFEB nuclear translocation. We also fund that in presence of VCP mutants, TFE3 activation is mediated by PPPC3B, a phosphatase triggered by increased cytoplasmic Ca^2+^, as occurs during LMP. This finding is intriguing because, so far, only TFEB expression was studied and reported to be altered in muscles of a transgenic VCP mouse model but at a very late stage of disease, and long after the beginning of when lysosomal damage occurred [37]. It would be of note to determine whether in these mice TFE3 is activated at the earliest stages, as well as if TFEB and TFE3 trigger alternative pathways.

Together, these data show an alteration of lysosome functional balance that may help to explain the presence of aberrant cytoplasmic vacuole-like structures in VCP patients affected tissues. In line with our data, studies in in vitro models related to VCP mutants show the presence of abnormal autophagolysosomes [21,73], which we demonstrated to correlate with lysosomal membrane rupture followed by the activation of the autophagic flux via TFE3. Therefore, in the first stages of the disease, the activation of the autophagic pathway induced in response to VCP mutants may serve as a protective mechanism against the presence of toxic damaged lysosomes and protein aggregation. Further studies are needed to confirm these data in motor neurons in vivo. VCP mutants have already been proven to alter the autophagic pathway in animal models. Therefore, it will be of interest to analyse the impact of lysosomal VCP-mutant induced damage on TFE3 regulation in these animal models or in induced pluripotent stem cells (iPSCs)-derived motor neurons and muscle cells. Moreover, the specific mechanisms that trigger VCP-mutant induced lysosomal damage have still to be identified. Further steps may include the analysis of the direct interaction of VCP with damaged lysosomes possibly via lysosome isolation, as well as the mechanism by which the pathway leading to PPP3CB/TEF3 activation is triggered and if this depends upon Ca^2+^ release after LMP. A deeper knowledge of these steps or of the triggering point of the damage may provide further targets for potential therapeutic approaches. Furthermore, our findings underline an important implication of VCP in counteracting SOD1 mutant pathological mechanisms such as aggregation or lysosome impairment. The testing of VCP modulation in SOD1 models could propose novel approaches also for ALS pathology. Of interest, this approach could be extended to other pathologies where lysosome alterations have been identified as Alzheimer’s disease and Huntington’s disease (HD) [56,57,59].

Collectively, our data highlight the importance of VCP in maintaining cellular homeostasis and may help to better define VCP mechanisms. This could open to a possible new pharmacological target to counteract degenerative diseases characterised by damaged lysosomes such as MSP/IBMPFD, ALS and FTD.

## Supplementary Material

supplementary

## Data Availability

The data that support the findings of this study are available from the corresponding author upon reasonable request.

## Key points

- VCP mutants trigger lysosomal damage and morphological alterations, with increased size, decreased acidification and impaired enzyme activity.
- VCP mutants enhance autophagic flux in a specific TFE3-dependent mechanism.
- VCP WT modulation decreases levels of chemically and biologically induced lysosomal damage.
- VCP WT and mutants decrease the insoluble levels of SOD1 mutant.
