# Supplementary material for: Pathogenic variants of Valosin-containing protein induce lysosomal damage and transcriptional activation of autophagy regulators in neuronal cells
Source: Neuropathol Appl Neurobiol. Author manuscript; Available in PMC 2023 Oct 20. (PMC10588520; doi:10.1111/nan.12818)
Supplement: supplementary [file NIHMS1862639-supplement-supplementary.pdf]

Supporting Information

Table 1 – Antibodies list

	WB/FTA analysis dilution	IF/STED analysis dilution
mouse anti-VCP #ab11433	1:1,000	
rabbit anti-histone H3 #ab1791	1:40,000	
mouse anti-FLAG #F1804	1:1,000	1:500
rabbit anti-SQSTM1/p62 #P0067	1:1,000	1:500
rabbit anti-MAP1LC3BA/B #L8919	1:1,000	1:500
rabbit anti-TFE3 #HPA023881	1:3,000	
rabbit anti-TFEB #A303-673A	1:3,000	
mouse anti-α-tubulin #T6199	1:3,000	
rabbit anti-SOD1 #ADI-SOD-100	1:1,000	1:200
rabbit anti-GAPDH #sc-25778	1:3,000	
mouse anti-6xHIS #MA1-21315	1:1,000	1:500
goat anti-rabbit IgG-HRP #111-035-003	1:10,000	
goat anti-mouse IgG-HRP #115-035-003	1:10,000	
goat anti-mouse 549 Alexa Fluor® #A11072		1:1,000
goat anti-rabbit 488 Alexa Fluor® #A11070		1:1,000

Table 2 – Plasmid list and used quantities

	pCDNA3	pFLAG-VCP WT	pFLAG-VCP R155H	pFLAG-VCP R191Q	p6xHIS-VCP WT	p6xHIS-VCP R155H	p6xHIS-VCP R191Q	pEGFP-LGALS3	pEGFP-N1-TFEB	pEGFP-N1-TFE3	pLAMP1-GFP	pSOD1 WT	pSOD1 G93A
Figure 1C	0.8µg	0.8µg	0.8µg	0.8µg	-	-	-	-	-	-	-	-	-
Figure 2A	0.5µg	0.5µg	0.5µg	0.5µg	-	-	-	-	-	-	-	-	-
Figure 2B	1µg	1µg	1µg	1µg	-	-	-	-	-	-	-	-	-
Figure 2C	1µg	1µg	1µg	1µg	-	-	-	-	-	-	-	-	-
Figure 2D	1µg	1µg	1µg	1µg	-	-	-	-	-	-	-	-	-
Figure 2E	0.3µg	-	-	-	0.3µg	0.3µg	0.3µg	-	-	-	0.2µg	-	-
Figure 2F	-	-	-	-	0.3µg	0.3µg	0.3µg	-	-	-	0.2µg	-	-
Figure 2G	1µg	1µg	1µg	1µg	-	-	-	-	-	-	-	-	-
Figure 2H	0.3µg	0.3µg	0.3µg	0.3µg	-	-	-	0.2µg	-	-	-	-	-
Figure 2I	-	-	-	-	0.3µg	0.3µg	0.3µg	0.2µg	-	-	-	-	-
Figure 2J	0.3µg	0.3µg	0.3µg	0.3µg	-	-	-	0.2µg	-	-	-	-	-
Figure 3A	0.3µg	0.3µg	0.3µg	0.3µg	-	-	-	0.2µg	-	-	-	-	-
Figure 3B	0.3µg	0.3µg	0.3µg	0.3µg	-	-	-	0.2µg	-	-	-	-	-
Figure 3C	1µg	1µg	1µg	1µg	-	-	-	-	-	-	-	-	-
Figure 3D	0.5µg	0.5µg	0.5µg	0.5µg	-	-	-	-	-	-	-	-	-
Figure 3E	0.5µg	0.5µg	0.5µg	0.5µg	-	-	-	-	-	-	-	-	-
Figure 4A	0.3µg	-	-	-	-	-	-	0.2µg	-	-	-	0.3µg	0.3µg
Figure 4B	0.3µg	-	-	-	-	-	-	0.2µg	-	-	-	0.3µg	0.3µg
Figure 4C	0.2µg	0.2µg	0.2µg	0.2µg	-	-	-	0.15µg	-	-	-	0.15µg	0.15µg
Figure 4D	0.5µg	0.5µg	0.5µg	0.5µg	-	-	-	-	-	-	-	0.5µg	0.5µg
Figure 4E	0.3µg	0.3µg	0.3µg	0.3µg	-	-	-	-	-	-	-	0.2µg	0.2µg
Figure 4F	0.3µg	0.3µg	0.3µg	0.3µg	-	-	-	-	-	-	-	0.2µg	0.2µg
Figure 5A	1µg	1µg	1µg	1µg	-	-	-	-	-	-	-	-	-
Figure 5F	1µg	1µg	1µg	1µg	-	-	-	-	-	-	-	-	-
Figure 5M	0.5µg	0.5µg	0.5µg	0.5µg	-	-	-	-	-	-	-	-	-
Figure 5N	0.5µg	0.5µg	0.5µg	0.5µg	-	-	-	-	-	-	-	-	-
Figure 5O	1µg	1µg	1µg	1µg	-	-	-	-	-	-	-	-	-
Figure 6A	0.2µg	0.2µg	0.2µg	0.2µg	-	-	-	-	-	-	-	-	-
Figure 6H	0.3µg	0.3µg	0.3µg	0.3µg	-	-	-	-	0.2µg	-	-	-	-
Figure 6I	0.3µg	0.3µg	0.3µg	0.3µg	-	-	-	-	-	0.2µg	-	-	-
Figure 7B	0.3µg	0.3µg	0.3µg	0.3µg	-	-	-	-	-	-	-	-	-
Figure 8A	0.5µg	0.5µg	0.5µg	0.5µg	-	-	-	-	-	-	-	0.5µg	0.5µg
Figure 8B	0.5µg	0.5µg	0.5µg	0.5µg	-	-	-	-	-	-	-	0.5µg	0.5µg
Figure 8C	0.3µg	0.3µg	0.3µg	0.3µg	-	-	-	-	-	-	-	0.2µg	0.2µg
Figure 8D	0.3µg	0.3µg	0.3µg	0.3µg	-	-	-	-	-	-	-	-	0.2µg

Table 3 – RNA Quantification of figure 7A

#	Sample ID	Nucleic Acid Conc.	Unit	A260	A280	260/280	260/230	Sample Type	Factor
1	NT siRNA	0,5554	µg/µl	13,886	7,103	1,96	2,21	RNA	40
2	NT siRNA	0,7354	µg/µl	18,386	9,376	1,96	2,32	RNA	40
3	NT siRNA	0,7654	µg/µl	19,134	9,7	1,97	2,22	RNA	40
4	NT siRNA	0,7658	µg/µl	19,146	9,762	1,96	2,22	RNA	40
5	PPP3CB siRNA	0,4431	µg/µl	11,077	5,801	1,91	2,19	RNA	40
6	PPP3CB siRNA	0,289	µg/µl	7,225	3,833	1,89	2,26	RNA	40
7	PPP3CB siRNA	0,3126	µg/µl	7,816	4,068	1,92	2,23	RNA	40
8	PPP3CB siRNA	0,376	µg/µl	9,399	4,881	1,93	2,07	RNA	40

Table 4 – RNA Quantification of figure 5O

#	Sample ID	Nucleic Acid Conc.	Unit	A260	A280	260/280	260/230	Sample Type	Factor
1	pCDNA3	0,5983	µg/µl	14,956	7,646	1,96	2,15	RNA	40
2	pCDNA3	0,6224	µg/µl	15,559	8,001	1,94	2,2	RNA	40
3	pCDNA3	0,557	µg/µl	13,924	7,103	1,96	1,86	RNA	40
4	pCDNA3	0,4293	µg/µl	10,732	5,551	1,93	1,94	RNA	40
5	VCP WT	0,5694	µg/µl	14,234	7,31	1,95	2	RNA	40
6	VCP WT	0,2806	µg/µl	7,014	3,655	1,92	1,97	RNA	40
7	VCP WT	0,4564	µg/µl	11,409	5,933	1,92	2,07	RNA	40
8	VCP WT	0,4455	µg/µl	11,139	5,82	1,91	2,11	RNA	40
9	VCP R155H	0,3591	µg/µl	8,977	4,682	1,92	2,07	RNA	40
10	VCP R155H	0,4387	µg/µl	10,968	5,617	1,95	1,77	RNA	40
11	VCP R155H	0,4333	µg/µl	10,831	5,428	2	1,6	RNA	40
12	VCP R155H	0,4607	µg/µl	11,517	5,936	1,94	2,07	RNA	40
13	VCP R191Q	0,1783	µg/µl	4,458	2,405	1,85	2,2	RNA	40
14	VCP R191Q	0,3821	µg/µl	9,553	5,049	1,89	2	RNA	40
15	VCP R191Q	0,1985	µg/µl	4,963	2,594	1,91	1,84	RNA	40
16	VCP R191Q	0,3078	µg/µl	7,694	4,075	1,89	2,09	RNA	40
